# Visualization of the Redox Status of Cytosolic Glutathione Using the Organelle- and Cytoskeleton-Targeted Redox Sensors

**DOI:** 10.3390/antiox9020129

**Published:** 2020-02-03

**Authors:** Yuta Hatori, Takanori Kubo, Yuichiro Sato, Sachiye Inouye, Reiko Akagi, Toshio Seyama

**Affiliations:** 1 Faculty of Pharmacy, Yasuda Women’s University, Hiroshima 731-0153, Japan; kubo-t@yasuda-u.ac.jp (T.K.); sato-y@yasuda-u.ac.jp (Y.S.); akagi@yasuda-u.ac.jp (R.A.); seyama@yasuda-u.ac.jp (T.S.); 2 Department of Pharmacy, Faculty of Pharmaceutical Sciences, Sanyo-Onoda City University, Yamaguchi 756-0884, Japan; inoues@rs.socu.ac.jp

**Keywords:** glutathione, organelle redox state, oxidative stress, reactive oxygen species, redox homeostasis

## Abstract

Glutathione is a small thiol-containing peptide that plays a central role in maintaining cellular redox homeostasis. Glutathione serves as a physiologic redox buffer by providing thiol electrons for catabolizing harmful oxidants and reversing oxidative effects on biomolecules. Recent evidence suggests that the balance of reduced and oxidized glutathione (GSH/GSSG) defines the redox states of Cys residues in proteins and fine-tunes their stabilities and functions. To elucidate the redox balance of cellular glutathione at subcellular resolution, a number of redox-sensitive green fluorescent protein (roGFP) variants have been developed. In this study, we constructed and functionally validated organelle- and cytoskeleton-targeted roGFP and elucidated the redox status of the cytosolic glutathione at a subcellular resolution. These new redox sensors firmly established a highly reduced redox equilibrium of cytosolic glutathione, wherein significant deviation was observed among cells. By targeting the sensor to the cytosolic and lumen sides of the Golgi membrane, we identified a prominent redox gradient across the biological membrane at the Golgi body. The results demonstrated that organelle- and cytoskeleton-targeted sensors enable the assessment of glutathione oxidation near the cytosolic surfaces of different organelle membranes.

## 1. Introduction

Glutathione plays a major role in protecting cells against oxidative insults, such as free radicals, reactive oxygen species (ROS), and hydrogen peroxide [1]. Oxidized lipid products and hydrogen peroxides are catalytically detoxified by glutathione peroxidases at the expense of glutathione [2,3] and oxidatively modified protein thiols are reduced by glutaredoxins, which also depend upon glutathione [4]. The ratio of oxidized and reduced glutathione (GSSG/GSH) serves as a quantitative measure of oxidative insult. Additionally, recent evidence suggests that oxidized glutathione reacts with protein thiols to induce oxidative protein modification (i.e., disulfide formation and/or glutathionylation) [5,6,7,8]. Therefore, maintaining the redox balance of cellular glutathione is essential for normal homeostasis.

Multiple techniques have been exploited to estimate the redox balance of cellular glutathione [9,10,11,12,13,14]. Classically, oxidized and reduced glutathione in cell lysate have been quantitatively analyzed by chromatography with various detection methods, including UV/vis-HPLC [15] and LC-MS [16]. Enzymatic assays can be performed using a spectrophotometer and tend to be more commonly used [17]. Based on electrochemical detection (ECD), GSH/GSSG-selective biosensors have been developed [18]. Although GSSG/GSH levels vary per cell and the ratio can be quantitatively determined by chromatographic analyses, redox potential (*E*_GSSG/GSH_) depends upon in situ concentrations of intracellular GSSG/GSH, which are difficult to measure accurately. Furthermore, it is highly challenging to distinguish between compartmentalized glutathione pools. To transcend these limitations, a series of genetically encoded redox sensors and fluorescent dyes have been developed and successfully applied for redox biology [19]. One of the most elegant and versatile designs of glutathione-specific sensors is glutaredoxin-1-redox-sensitive green fluorescent protein 2 (Grx1-roGFP2) invented by Gutscher et al. [11]. Fluorescent sensors enable measurement of *E*_GSSG/GSH_ in living cells and allow visualization of the previously unseen redox dynamics of intracellular glutathione [11,13,19,20,21].

We were interested in whether the cytosol maintains a uniform redox balance of glutathione [22]. Cells harbor different organelles and structures capable of physically and chemically affecting the proximal environment. Assuming local redox effects of various intracellular structures, even the cytoplasmic milieu might exhibit an inconsistent pattern of glutathione oxidation. For example, NADPH oxidases (Noxs) produce H_2_O_2_ and are present at the biological membranes of specific organelles [23]. Induction/activation of Nox proteins can increase local concentrations of H_2_O_2_ around the protein. Therefore, in our previous study, we developed a membrane-targeted derivative of Grx1-roGFP2 [22] that was successfully attached to the cytosolic surfaces of biological membranes, including the plasma membrane (PM) and intracellular vesicles, enabling the assessment of local information concerning cytosolic glutathione. Our analysis demonstrated that glutathione was highly reduced near the PM while relatively oxidized near some types of intracellular structures. These findings revealed that the redox balance of the glutathione pair is not completely uniform within the cytosol; therefore, the detailed redox pattern remains to be elucidated.

To investigate the spatial pattern of glutathione oxidation within the cytosol, we further developed a set of Grx1-roGFP2 derivatives targeted to the cytoskeleton and the cytosolic sides of various organelles. In HeLa cells, the redox state of glutathione is reduced near actin and keratin filaments and cytosolic surfaces of ER, peroxisomes, and plasma membranes, which collectively reflect the overall reduced state of the whole cytosol. Interestingly, we detected a significant level of oxidation around the Golgi membrane, which seemingly coincides with marked oxidation in the Golgi lumen. These findings provide insight into the previously unseen redox landscape of cytosolic glutathione and highlight the unique redox feature of the Golgi membrane.

## 2. Materials and Methods 

### 2.1. Plasmid Construction

Plasmid pEIGW/Grx1-roGFP2 [11] was gifted from Dr. Tobias Dick (Addgene plasmid #64990; Addgene, Watertown, MA, USA) [11] and used as template DNA for amplifying Grx1-roGFP2 cDNA. Plasmids encoding organelle-targeted fluorophores were kind gifts from the following investigators: mCh-Sec61β from Dr. Gia Voeltz (Addgene#49155; Addgene) [24]; pmScarlet_Giantin_C1, pmTurquoise2-Tubulin, and pmTurquoise2-Golgi from Dr. Dorus Gadella (Addgene#85048, #36202, and #36205; Addgene) [25,26]; mCherry-Lifeact-7 and mCherry-PMP-N-10 from Dr. Michael Davidson (Addgene plasmid #54491 and #55120; Addgene); and pKeratin-miRFP703 from Dr. Vladislav Verkhusha (Addgene plasmid # 79990; Addgene) [27]. DNA primers used in the study are listed in Supplementary Data 1 and the procedures used for plasmid construction are fully documented in Supplementary Data 2. Briefly, cDNA of Grx1-roGFP2 was amplified using Pfu Ultra DNA polymerase (Agilent Technologies, Santa Clara, CA, USA). The fluorophore sequence in each organelle-targeted construct was replaced with the amplified DNA fragment to produce an organelle-targeted version of Grx1-roGFP2 and the DNA sequences of all amplified regions were verified. Construction of palmitoyl Grx1-roGFP2 (Palm-Grx1-roGFP2) was previously described [22]. Plasmid DNA with transfection-compatible purity was prepared using a Midi kit (Qiagen, Hilden, Germany) and quantified by spectrophotometer Nanodrop-1000 (Thermo Fisher scientific, Waltham, MA, USA).

### 2.2. Cell Culture and Transfection

HeLa cells were gift from Dr. Shigeru Sassa (The Rockefeller University, New York, NY, USA) and maintained in Dulbecco’s minimal essential medium (DMEM) supplemented with 10% (v/v) fetal bovine serum (FBS; Thermo Fisher scientific), 100 U/mL penicillin, and 100 µg/mL streptomycin. Cells were cultured on collagen-coated 35-mm FluoroDishes (FD35-100; World Precision Instruments, Inc., Sarasota, FL, USA). Upon reaching the sub-confluent stage, cells were transfected with 2 μg of plasmid DNA using Lipofectamine 3000 (Thermo Fisher scientific, Waltham, MA, USA) according to manufacturer’s instruction. Culture medium was replenished 24 h after transfection and redox assays were performed typically 48 h after transfection.

### 2.3. Quantitative Analysis of Glutathione Oxidation

Glutathione oxidation was quantitatively assessed using a previously documented method [22]. Live cells on FuluoroDishes were directly analyzed by confocal microscopy. For obtaining high-resolution images, cells on glass cover slips were treated with 10 mM N-ethylmaleimide (NEM) for 10 min at room temperature prior to paraformaldehyde (PFA) fixation. This ‘redox fixation’ preserves redox status of the sensors as in a live cell [6,22]. Fixed samples were mounted with 4-(2-hydroxyethyl)-1-piperazineethanesulfonic acid (HEPES)-buffered glycerol medium (10% glycerol, 10 mM 4-(2-hydroxyethyl)-1-piperazineethanesulfonic acid, pH 7.4) on a glass slide and sealed. Images were obtained using a confocal microscope (FV1000; Olympus, Tokyo, Japan). Two distinct excitation wavelengths (488 and 405 nm) were used for 2-channel imaging. The emission wave length was set to 540–560 nm. The confocal microscope system was equipped with a gallium arsenide phosphide (GaAsP) PMT detector unit (Olympus, Japan). The ratio of the intensities of the two channels was designated as RI405/I488, which served as a measure of sensor oxidation.

For testing sensor reactivity, cellular glutathione was forcibly oxidized by adding hydrogen peroxide (H_2_O_2_) and reversed by dithiothreitol (DTT), a widely-used cell-permeable reductant [28]. Concentrations are indicated in each figure. The change of redox status of a sensor was monitored by time-lapse confocal microscopy.

All images were processed using ImageJ software (National Institutes of Health, Bethesda, MD, USA) [29] with the Fiji package [30]. 

### 2.4. Immunostaining

Cells were placed on cover glasses in a 12-well plate. Cells were treated with 3.7% PFA dissolved in phosphate-buffered saline (PBS) for 20 min, followed by permeabilization using 0.2% (w/v) Triton X-100 in PBS. After detergent treatment for 10 min, cells were then incubated in 1% (w/v) BSA/1% (w/v) gelatin for 30 min. Immunostaining was performed using antibodies listed in Supplementary Data 3. Immunostained samples were mounted on slide glasses using Fluoromount-G with DAPI (Southern Biotech, Birmingham, AL, USA).

## 3. Results

### 3.1. Targeting Redox Sensors to the Cytoplasmic Sides of Various Organelle Membranes

**Figure 1:**
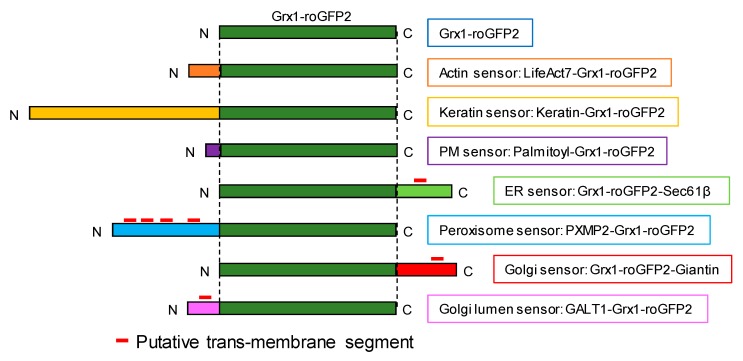
Construction of glutathione oxidation sensor (Grx1-roGFP2) targeted to the cytoplasmic sides of various organelle membranes and cytoskeletons. Grx1-roGFP2 sequence (green box) were fused with exogenous sequences in specific membrane topologies so that the sensor faced to the cytosolic side. GALT1-Grx1-roGFP2 was designed to orient the sensor domain to the lumen. Transmembrane segments were predicted by SOSUI [32] and are indicated by red lines.

We sought to analyze cytosolic glutathione using the recently developed biosensor, Grx1-roGFP2 [11]. To apply the established sensor for assessing intracellular patterns of glutathione redox status, it was critical that the free cytosolic form of Grx1-roGFP2 was diffused within the cytosol, potentially resulting in the mixing of overall cytosolic information. To stabilize the sensor to specific locations within the cytosol, we expressed the sensor as various fusion proteins (Figure 1). As cytoskeleton-targeted sensors, Grx1-roGFP2 was fused with either LifeAct [31] or keratin. To target the sensor to the cytoplasmic sides of organelle membranes, Grx1-roGFP2 was fused with either Sec61β (endoplasmic reticulum; ER), peroxisomal membrane protein 2 (PXMP2; peroxisome), or giantin (Golgi body), in addition to a previously reported guanine nucleotide-exchange factor (GEF)-fused version (in the PM). For direct comparison between the two sides of a biological membrane, a Golgi lumen-targeted sensor was also developed by fusing galactosyltransferase 1 (GALT1; Golgi body).

**Figure 2:**
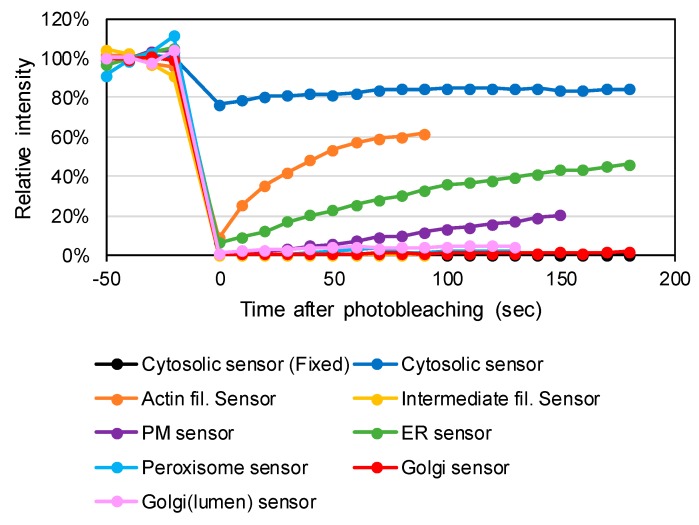
Fluorescence recovery after photobleaching (FRAP) confirmed effective anchoring of Grx1-roGFP2 to organelle membranes or cytoskeletons. To assess association to the cytoskeletons and organelle membranes, fluorescence recovery was monitored for 180 s after photobleaching. Original confocal images are shown in Supplementary Data 4. As non-diffusible controls, cells were fixed with 3.7% paraformaldehyde prior to FRAP (Grx1-roGFP2 Fixed).

To confirm stabilization to the cytoskeletons or biological membranes, we performed fluorescence recovery after photobleaching (FRAP) experiments. Soluble cytosolic proteins are freely diffused while the motion of membrane-bound proteins is highly limited, exhibiting slow FRAP kinetics [33]. Constructed plasmids were transfected into HeLa cells. As a static control, Grx1-roGFP2 was fixed with PFA prior to the FRAP assay. This control sample showed irreversible fluorescence quenching (dashed box in Supplementary Data 4). Non-fixed Grx1-roGFP2 was characterized by immediate fluorescence recovery, which was hardly detected due to the duration of image scanning (Supplementary Data 4 and Figure 2). By contrast, the PM-targeted sensor (Palmitoyl-Grx1-roGFP2) was quenched only within the exposed area and the fluorescence was slowly recovered with a time constant of 300 s (Figure 2). Time courses of the photo recoveries of roGFP-fusion versions of the sensors are shown in Figure 2. The t_1/2_ value for the ER-targeted sensor (Sec61β-Grx1-roGFP2) was 180 s, whereas that for the actin filament-targeted sensor (LifeAct-Grx1-roGFP2) exhibited a relatively higher diffusion rate (t_1/2_ = 30 s), which was still much slower than Grx1-roGFP2. 

Additionally, the diffusion rate was much slower than the previously determined reaction rate for Grx1-roGFP2 [11]. Therefore, we determined that the signal from the actin filament-targeted sensor (LifeAct-Grx1-roGFP2) reflected local information rather than an average value within actin filaments. The Golgi- and peroxisome-targeted sensors (Grx1-roGFP2-giantin, GALT1-Grx1-roGFP2 and PXMP2-Grx1-roGFP2) did not show fluorescence recovery, indicating complete stabilization to the organelle membranes.

**Figure 3:**
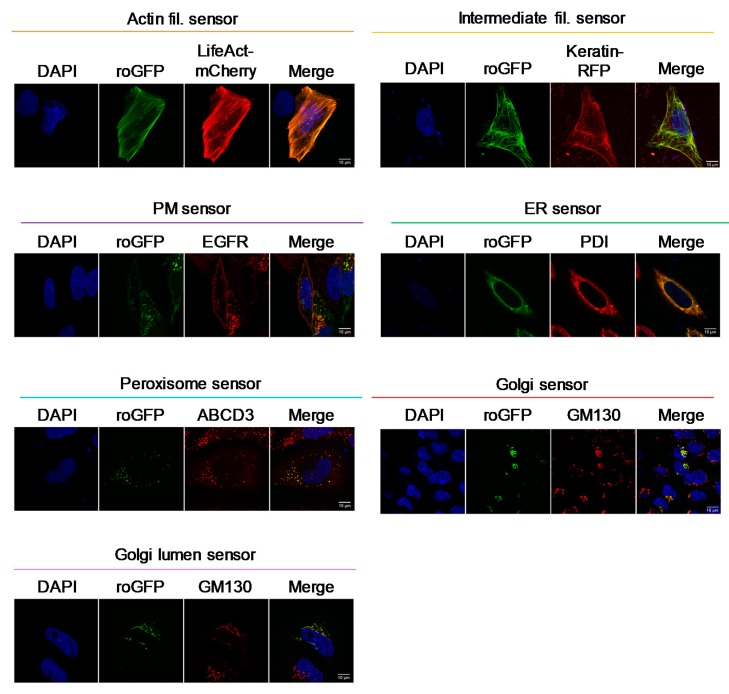
Successful targeting of Grx1-roGFP2 to the cytoplasmic sides of various organelle membranes. DNA constructs were transfected into HeLa cells and protein localization was analyzed by immunostaining for organelle markers; GM130 at the Golgi membrane, epidermal growth factor receptor (EGFR) at the plasma membrane, ABCD3 at peroxisome, and protein disulfide isomerase (PDI) at endoplasmic reticulum. LifeAct-mCherry and Keratin-mCherry were used as markers of actin and intermediate filaments. Scale bars, 10 µm.

Subcellular localizations of the constructed sensors were determined by either co-immunostaining with various organelle markers or co-expression with marker proteins. As shown in Figure 3, correct localizations were observed for all sensors.

### 3.2. Testing the Redox Responses of the Developed Sensors

**Figure 4:**
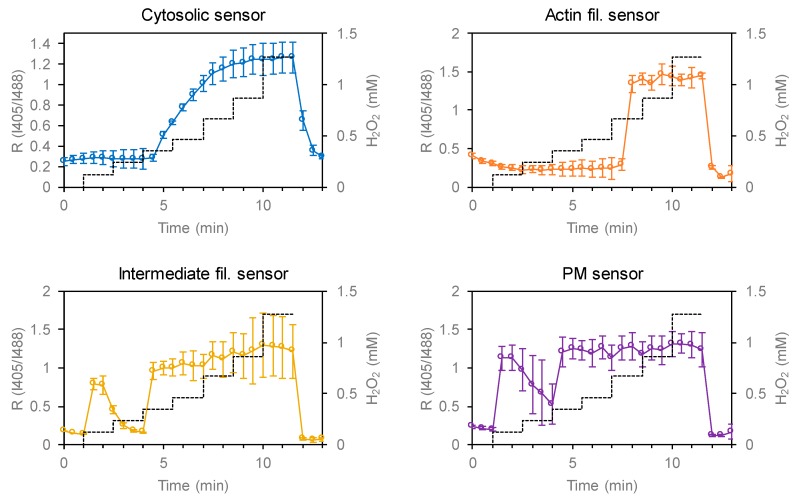

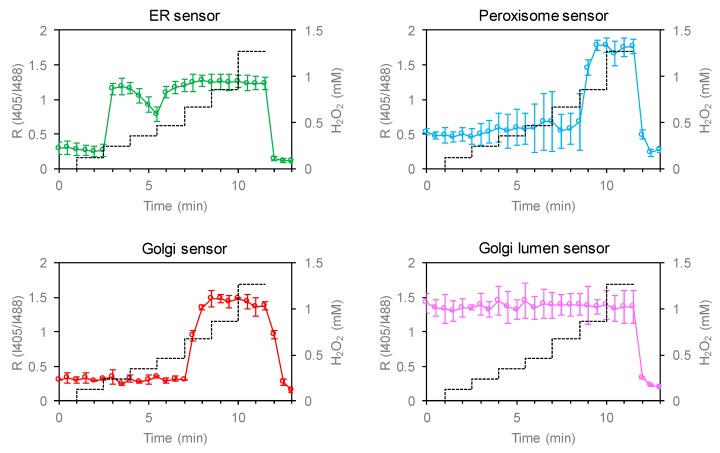
Validation of redox reactivity of constructed sensors in HeLa cells. Redox reactivity was tested by H_2_O_2_ titration. I405 and I488 images were taken by confocal microscopy and the ratio R_I405/I488_ was obtained for each cell present within the observed area (typically n > 5). Dashed lines indicate concentrations of H_2_O_2_. Sensor oxidation was reversed by adding 5 mM dithiothreitol (DTT) at time point of 10.5 min.

**Figure 5:**
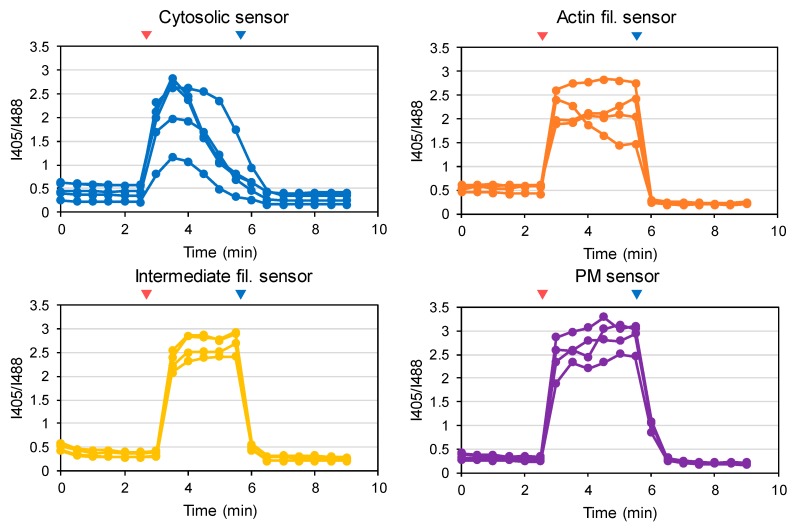

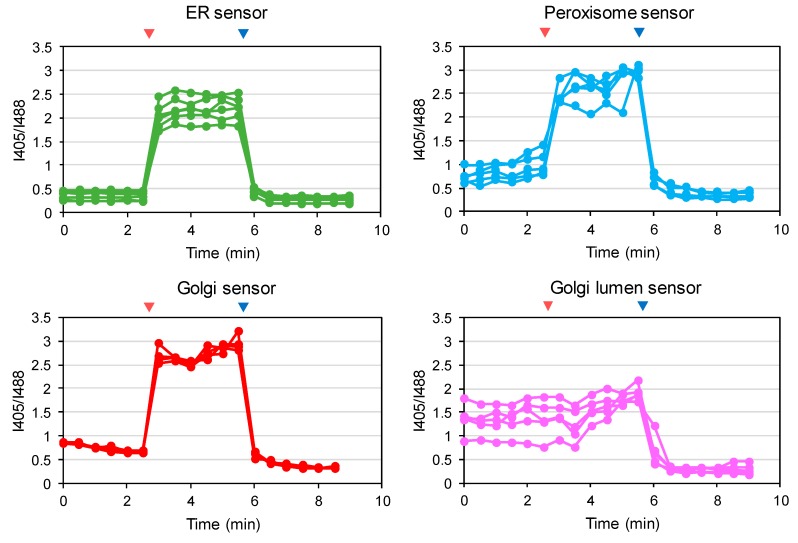
Validation of the redox reactivity of constructed sensors in HeLa cells. Reversible redox modulation was confirmed by sequential treatment with H_2_O_2_ (oxidant, red arrow heads) and 5 mM DTT (reductant, blue arrow heads). Based on the results of H_2_O_2_ titration, actin and peroxisome sensors were tested by 2 mM H_2_O_2_ while others were assayed with 0.5 mM H_2_O_2_. Blue and red arrow heads represent the time of adding H_2_O_2_ and DTT, respectively.

In order to test the reactivity of the developed sensors, glutathione oxidation was induced by adding H_2_O_2_ to the culture medium (Figure 4 and Figure 5). To check the reversibility of sensor oxidation, cells were subsequently treated with dithiothreitol. Glutathione oxidation by H_2_O_2_ depends on enzymatic processes and may very kinetically among different parts of a cell. Considering possible differences in H_2_O_2_ dependence, we performed a titration assay where H_2_O_2_ concentration was increased stepwise above 1 mM. Fluorescence (λ_ex_ 405 and 488 nm) from individual cells were quantified by confocal microscopy and sensor oxidation was calculated according to the ratio of signals obtained by excitation at 405 and 488 nm (R_I405/I488_). All sensors showed normal redox responses, indicating intact responses by these sensors. At low H_2_O_2_ concentrations (<200 μM), glutathione was only transiently oxidized and reduced back toward resting state, which may reflect a cellular antioxidant reaction. To capitulate the whole antioxidant system, at least 0.5–1 mM H_2_O_2_ was required depending on the location within the cytosol. The initial I405/I488 values for the Golgi lumen-targeted sensor were significantly higher than those of other sensors, suggesting that the lumen of Golgi bodies were constitutively oxidized (details are provided in the following section). The maximal and minimal R_I405/I488_ values for all sensors were comparable to that of Grx1-roGFP2, suggesting that the same parameters can be applied for estimating redox potentials. We further assessed possible pH effect on the spectral characteristics of Grx1-roGFP2. Cytosolic pH was artificially changed to 7.5, 6.5, 5.5, or 4.5 using ionophores and R_I405/I488_ value was monitored by live cell imaging, demonstrating that the signal from Grx1-roGFP2 is stable within the tested pH range (Supplementary Data 5).

### 3.3. Unique Redox Environment near Organelle Membranes and Transmembrane Redox Gradient

**Figure 6:**
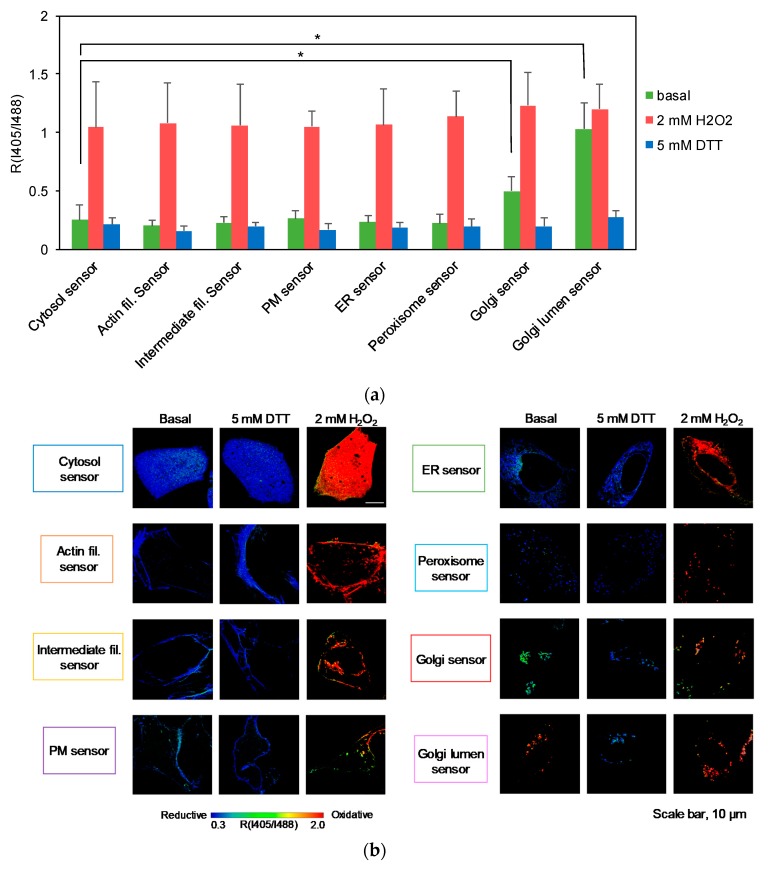
Membrane-targeted sensors reported overall reduced E_GSSG/GSH_ levels in organelle-peripheral cytoplasm. Marked redox gradient across Golgi membrane was observed. (**a**) HeLa cells expressing redox sensors were subjected to ‘redox fixation’ (see Materials and Methods for the detail) and I405/I488 ratiometric imaging was performed by confocal microscopy. R_I405/I488_ value represents the degree of sensor oxidation. As an oxidized/reduced control, cells were treated with either 2 mM H_2_O_2_ (oxidized) or 5 mM DTT (reduced) for 2 min prior to redox fixation. Data represent the average of multiple cells (n > 20) and standard deviation. Asterisks indicate significant differences assessed by student’s t-test (* *p* < 0.05). (**b**) Representative R_I405/I488_ ratiometric images are shown. Scale bars, 10 μm. The original I405 and I488 images are shown in Supplementary Data 7.

We then analyzed the steady state redox status of HeLa cells at subcellular resolution using the various fusion versions of Grx1-roGFP2. At high magnification/resolution, even minute (submicron) movements of intracellular structures become a critical problem for generating ratiometric images. To avoid this, the cell fixation method was used. Indeed, superimpositions of I405 and I488 images showed that cell structures were completely static in these samples (Supplementary Data 6). Prior to fixation, cells were incubated with Cys-labeling reagents in order to protect the sensors from spontaneous oxidation (details are documented in Materials and Methods section). Using this method, live cell redox states were successfully preserved; live cell oxidation and reduction were successfully preserved in H_2_O_2_- and DTT-treated controls (Figure 6 and Supplementary Data 7).

R_I405/I488_ values were measured from more than 20 cells for each sensor and are summarized in Figure 6a. As noted, all sensors, except for Golgi-targeted sensors, returned markedly low R_I405/I488_ values, indicating that the cytosolic sides of the PM, ER, peroxisome membranes, as well as regions proximal to actin and keratin filaments were highly reduced (Figure 6a). Interestingly, the Golgi sensor reported an R_I405/I488_ value significantly higher than other sensors, suggesting the possibility that the oxidation level of cytosolic glutathione is specifically high near Golgi membranes. The Golgi lumen exhibited saturated level of sensor oxidation, demonstrating a prominent redox gradient across the Golgi membrane. The same trend was obvious on high resolution/magnification ratiometric images (Figure 6b). Considering the possibility that the fluorophores were oriented to the wrong sides of the membrane due to topological misalignments, Grx1-roGFP2-Giantin and GALT1-Grx1-roGFP2 were further subjected to a fluorescence protease protection (FPP) assay using digitonin as the plasma membrane-permeable detergent [34]. Grx1-roGFP2-Giantin was more labile to protease digestion compared to GALT1-Grx1-roGFP2, suggesting that these sensors were oriented to the cytosol and the lumen, respectively (Supplementary Data 8). These results firmly established that, even in basal conditions, the levels of glutathione oxidation are not completely the same within the cytosol and that membrane-proximal regions are capable of maintaining a unique redox equilibrium significantly different from the rest of the cytosol.

## 4. Discussion

Cells utilize a system to maintain their redox environment and equilibration within a specific redox range allows proteins and biomolecules within this environment to maintain their proper redox status [1]. Glutathione constitutes the major thiol component within a cell and serves as a noncatalytic redox buffer. Additionally, multiple antioxidant enzymes utilize glutathione as a substrate or cofactor [35]. Grxs harbor a Cys-X-X-Cys canonical redox-active motif that promotes the transfer of electrons between glutathione and protein thiols. The thiol-disulfide exchange, mediated by Grxs, is reversible and eventually reaches an equilibrium [4]. In this sense, the function of a Grx can be defined as a “pipe” that kinetically connects two distinct thiol pools (i.e., glutathione and protein thiols). Consequently, the redox status of a protein thiol is governed by the redox potential of the glutathione pool (E_GSSG/GSH_). 

The redox environment within the cell is spatially heterogeneous, which is evident from previous analyses using organelle-targeted redox sensors [20,36]. The mitochondrial intermembrane space is characterized by a higher redox potential relative to that in the cytosol and reflects mitochondrial activity involved in ROS generation [37]. Moreover, the ER lumen reportedly has an oxidative E_GSSG/GSH_ value of about −0.21 V [12], which exceeds the average value of the cytoplasmic pool (−3.0 V) [21]. 

Although the redox states of organelle lumens have been explored, potential heterogeneity within the cytosol had not been formally assessed until our previous study using a membrane-anchored version of Grx1-roGFP2 [22]. Assuming that sensor diffusion within the cytosol results in a critical decline of spatial resolution during analysis, we stabilized the sensor to the cytosolic sides of biological membranes. The original cytosolic sensor reported an E_GSSG/GSH_ value of −330 mV, whereas the PM- and vesicle-anchored sensors reported −275 and −256 mV, respectively, clearly demonstrating that the redox potential of cytosolic glutathione is highly heterogeneous [22]. To illuminate the redox landscape of cytosolic glutathione, we created 7 constructs with various targeting-fusion domains, including keratin (intermediate filaments), LifeAct (actin filaments), Sec61β (ER membrane), PXMP2 (peroxisomal membrane), Giantin (Golgi membrane), GALT1 (Golgi membrane), and a previously constructed GEF5 fusion (palmitoylation motif; PM and intracellular vesicles). These fusion proteins were successfully expressed and targeted to the locations of interest. Except for the Golgi sensors, the above 5 sensors, as well as the original Grx1-roGFP2, reported markedly low levels of glutathione oxidation, which were comparable to the DTT-treated control cells, demonstrating that the cytosol is indeed a highly reductive environment. The Golgi-targeted sensors revealed two intriguing facts: (i) that the Golgi membrane forms a steep redox gradient between cytosol and lumen sides and (ii) that the cytosolic surface of the Golgi membrane exhibits a relatively high level of glutathione oxidation compared to other organelle membranes and cytoskeletons tested in this study (PM, ER, peroxisome, actin, and keratin). The corresponding Eh values of the original sensor and Grx1-roGFP2-Giantin were estimated to be −295 and −277 mV, respectively. Beside the average redox trends among intracellular compartments, it is clear that the redox status is substantially different between distinct cells (Figure 4, Figure 5, and Figure 6a). Imaging-based analysis using Grx1-roGFP2 derivatives is capable of distinguishing cell-to-cell variation and, in this regard, has a clear advantage over other analytical approaches.

The oxidative source for both sides of the Golgi membrane has not yet been determined. We preliminarily expected the involvement of membrane-bound Noxs, which are also present on the Golgi membrane (Nox2) [38]. It is possible that a Nox inhibitor, such as VAS2870, might modulate the redox status of the Golgi membrane, which is a currently ongoing experiment. As an alternative mechanism, the luminal GSSG pool might affect the cytosolic side across biological membranes. In yeast, oxidized glutathione is rapidly transported into the vacuole by the ATP-binding cassette (ABC) transporter Ycf1 [39]. A similar process in mammalian cells, if any, might accumulate oxidized glutathione in the secretory vesicles, pointing to a bold speculation that potential back-leakage of oxidized glutathione might, in turn, affect the Golgi-peripheral regions.

Glutathione oxidation near the Golgi body raises questions regarding the functional consequences. Elevation of E_GSSG/GSH_ is associated with oxidative modification of Cys residues in various proteins, including the copper-handling chaperone Atox1 [5,6,40] and transporters ATP7A and ATP7B [7,41]. The copper-binding sites of these proteins are formed by Cys residues and hampered by thiol oxidation, eventually decreasing the activity of copper transport into the secretory pathway [5,6]. It is tempting to speculate that the activities of Golgi-resident proteins might be tuned depending on the calibration of local E_GSSG/GSH_ levels. Ryanodine receptor type 1 channels, a Ca-releasing channel at the sarcoplasmic reticulum (SR), was reported to be redox-regulated depending on E_GSSG/GSH_ [42], which points to an intriguing question as to how E_GSSG/GSH_ is maintained on the surface of the SR membrane. Our sensor, Grx1-roGFP2-sec61β, is highly expected to be targeted to rough ER membranes and an SR-targeted sensor might report a different result. To this end, SR-associating peptides, such as phospholamban and sarcolipin, may be promising candidates of signal sequences for targeting the sensor to SR membranes. These sensors are, in principle, applicable to a wide range of cell types as far as the sensor genes can be delivered into the cells. Particularly, some pathologic conditions, such as inflammatory bowel disease [43,44] and Parkinson’s disease [45], have been associated with glutathione oxidation and application of the Grx1-roGFP2 derivatives to in vitro pathologic models may bring new information regarding the redox modulation during pathogenesis.

## 5. Conclusions

This study provided new derivatives of redox sensors targeted to various positions of the cytosol, revealing the redox heterogeneity of the cytosolic glutathione at a subcellular resolution. The findings highlighted the significance of redox heterogeneity within the cytosol.

## Supplementary Materials

The following are available online at https://www.mdpi.com/2076-3921/9/2/129/s1, Supplementary Data 1: Primers, restriction enzymes, and template plasmids used for constructing organelle-targeted Grx1-roGFP2; Supplementary Data 2: Subcloning procedures; Supplementary Data 3: Antibodies; Supplementary Data 4: Original data of fluorescence recovery after photobleach (FRAP) analyses; Supplementary Data 5: Analyses of Grx1-roGFP2 at different intracellular pH; Supplementary Data 6: Superimposition of I405 and I488 channels; Supplementary Data 7: Representative high-resolution images of fixed cells expressing Grx1-roGFP2 derivatives; and Supplementary Data 8: Original I405 and I488 images of fixed cells expressing Grx1-roGFP2 derivatives.
