# Supplementary material for: Visualization of the Redox Status of Cytosolic Glutathione Using the Organelle- and Cytoskeleton-Targeted Redox Sensors
Source: Antioxidants (Basel). 2020 Feb 3;9(2):129. doi: 10.3390/antiox9020129 (PMC7070464; doi:10.3390/antiox9020129)
Supplement: Supplementary file 1 [file antioxidants-09-00129-s001.pdf]

Supplementary Data 1: Primers, Restriction enzymes, template plasmids used for constructing organelle-targeted Grx1-roGFP2

Oligonucleotides, Restriction enzymes, template plasmids used for constructing organelle-targeted Grx1-roGFP2

Construct name	Oligonucleotide	sequence	Restriction enzyme	Original Plasmid
Subcloning strategy I				
LifeAct7-Grx1-roGFP2	LifeAct_F	ttGGATCCaccggtcgccaccatggctcaagagtttgtgaactg	BamHI	mCherry-Lifeact-7
	LifeAct_R	ttGCGGCCGCttacttgtacagctcgtccatgccgag	NotI	
GALT1-Grx1-roGFP2	GALT1_F	ttGGATCCaccggtcgccaccatggctcaagagtttgtgaactg	BamHI	pmTurquoise2-Golgi
	GALT1_R	ttGCGGCCGCttacttgtacagctcgtccatgccgag	NotI	
Keratin-Grx1-roGFP2	Keratin_F	ttGGTACCgccggtcgccaccatggctcaagagtttgtgaactg	KpnI	pKeratin-miRFP703
	Keratin_R	ttGCGGCCGCttacttgtacagctcgtccatgccgag	NotI	
PXMP2-Grx1-roGFP2	PXMP2_F	ttACCGGTcgccacatggctcaagagtttgtgaactg	AgeI	mCherry-PMP-N-10
	PXMP2_R	ttGCGGCCGCttacttgtacagctcgtccatgccgag	NotI	
Subcloning strategy II				
Grx1-roGFP2	Grx1roGFP2_F	ttAAGCTTcaccatggctcaagagtttgtgaac	HindIII	pEIGW/Grx1-roGFP2
	Grx1roGFP2_R	ttGCGGCCGCccttgtacagctcgtccatgccgag	NotI	
Grx1roGFP2_Sec61b	Sec61b_F	ttGCGGCCGCagatctatgcctggtccgac	NotI	mCh-Sec61β
	Sec61b_R	ttTCTAGActcgaacgagtgtacttgccccaaatg	XbaI	
Grx1roGFP2_Giantin	Giantin_F	ttGCGGCCGCtccggactcagatctcgagg	NotI	pmScarlet_Giantin_C1
	Giantin_R	ttTCTAGActatagatggcccgtaaaac	XbaI	

Supplementary Data 2: Subcloning procedures

For constructing a series of targeted Grx1-roGFP2, two alternative strategies, namely, strategy I and II were adopted. LifeAct7-Grx1-roGFP2, GALT1-Grx1-roGFP2, Keratin-Grx1-roGFP2, and PXMP2-Grx1-roGFP2 were constructed by strategy I. Grx1-roGFP2, Grx1-roGFP2-Sec61β and Grx1-roGFP2-Giantin were constructed using strategy II.

In strategy I, fluorescent protein (FP) coding regions in the original plasmids were replaced by cDNA of Grx1-roGFP2. First, cDNA of Grx1-roGFP2 was amplified using Pfu Ultra DNA polymerase (Agilent Technologies, Santa Clara, CA, USA). pEIGW/Grx1-roGFP2 was used as DNA template and oligonucleotide primers were listed in Supplementary Data 1. Purified DNA fragments were cleaved by restriction enzymes as indicated in the list. Corresponding original plasmids coding organelle-targeted fluorophores were similarly cleaved so that fluorophore sequences were removed. Resulting DNA fragments were ligated and transformed into Escherichia coli strain XL10 gold (Agilent Technologies). After selection by antibiotics (either 50 μg/ml kanamycine or 100 μg/ml ampicilline), transformed bacteria was isolated and propagated, followed by plasmid purification using a Midi kit (Qiagen, Hilden, Germany).

In strategy II, cDNA of Grx1-roGFP2 was amplified by PCR using Pfu Ultra DNA polymerase (Agilent Technologies), pEIGW/Grx1-roGFP2 as template, oligonucleotide primers Grx1-roGFP2_F and Grx1-roGFP2_R (Supplementary Data 1). Purified DNA fragment was cleaved by HindIII and NotI. pCDNA3.1(+)/Myc-His vector (Thermo Fisher scientific) was cleaved by the same pair of restriction enzymes. Resulting DNA fragments were ligated and transformed into Escherichia coli strain XL10 gold (Agilent Technologies). After selection by 100 μg/ml ampicilline, transformed bacteria was isolated and propagated, followed by plasmid purification using a Midi kit (Qiagen, Hilden, Germany). The resulting

construct is named as pCDNA3.1(+)/Grx1-roGFP2-Myc-His, which was used as a cytosolic or freely diffused version of Grx1-roGFP2 in the study. In order to target Grx1-roGFP2 to specific organelles, fusion proteins were constructed using the following procedure. A partial sequence from Sec61β or Giantin was amplified using oligonucleotide primers and DNA templates shown in Supplementary Data 1. Amplified PCR products and pCDNA3.1(+)/Grx1-roGFP2-Myc-His were cleaved by restriction enzymes as indicated in the list. Resulting DNA fragments were ligated. For Giantin-fused version, a stop codon was included in the reverse primers to exclude Myc-His epitopes from the coding frame. Ligated DNAs were transformed into *Escherichia coli* strain XL10 gold (Agilent Technologies). After selection by 100 µg/ml ampicilline, transformed bacteria was isolated and propagated, followed by plasmid purification using a Midi kit (Qiagen, Hilden, Germany).

Quantity and purity of plasmid DNA was assessed by spectrophotometer Nanodrop (Thermo Fisher scientific, Waltham, MA, USA). Amplified DNA regions were fully sequenced.

Supplementary Data 3: Antibodies

Antibodies used in the study

antibody name	host animal	manufacturer	catalogue number
Primary antibodies			
anti-EGFR	Mouse, Monoclonal	Thermo Fisher	MA5-13070
anti-GM130	Rabbit, Monoclonal	Abcam	ab52649
anti-PDI	Mouse, Monoclonal	Abcam	ab2792
anti-ABCD3	Mouse, Monoclonal	ATLAS antibodies	AMAB91170
Secondary antibodies			
Alexa Fluor 568 Anti-mouse IgG(H+L)	Goat, Polyclonal	Life Technologies	A11004
Alexa Fluor 568 Anti-rabbit IgG(H+L)	Goat, Polyclonal	Life Technologies	A11011

Supplementary Data 4: Original data of Fluorescence recovery after photobleach (FRAP) analyses.

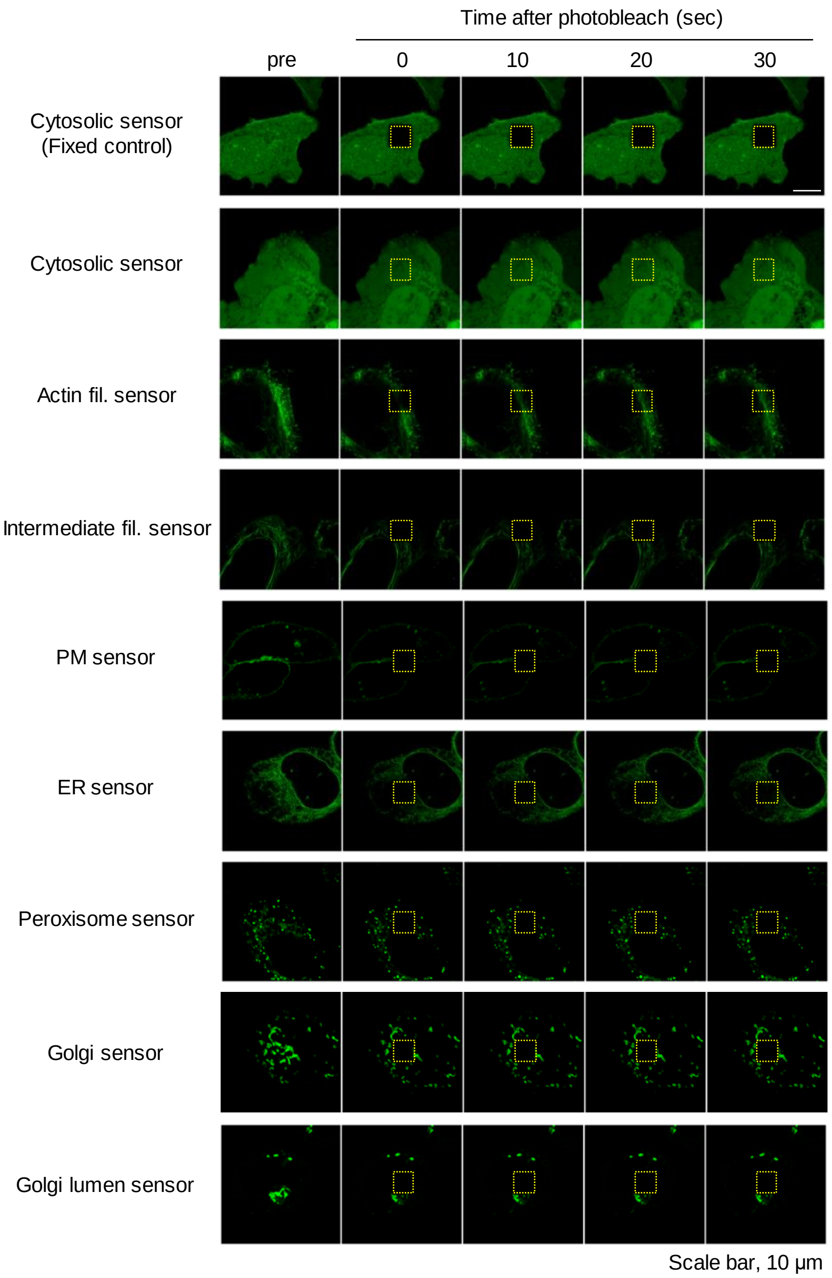

Photo-bleached areas are indicated by *yellow dashed* squares. Time-lapse images were taken for 180 sec after breaching. Images during the initial 30 sec are shown.

Supplementary Data 5: Analyses of Grx1-roGFP2 at different intracellular pH.

A

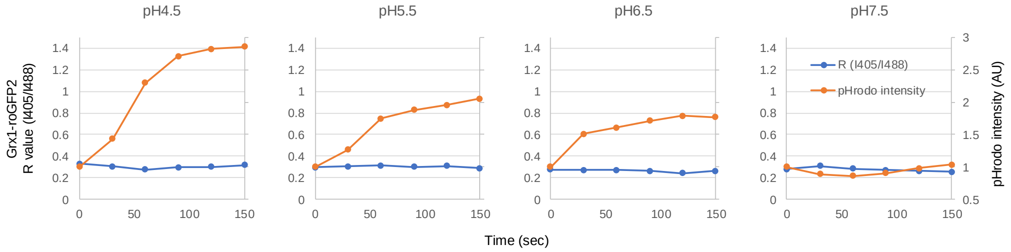

B-1

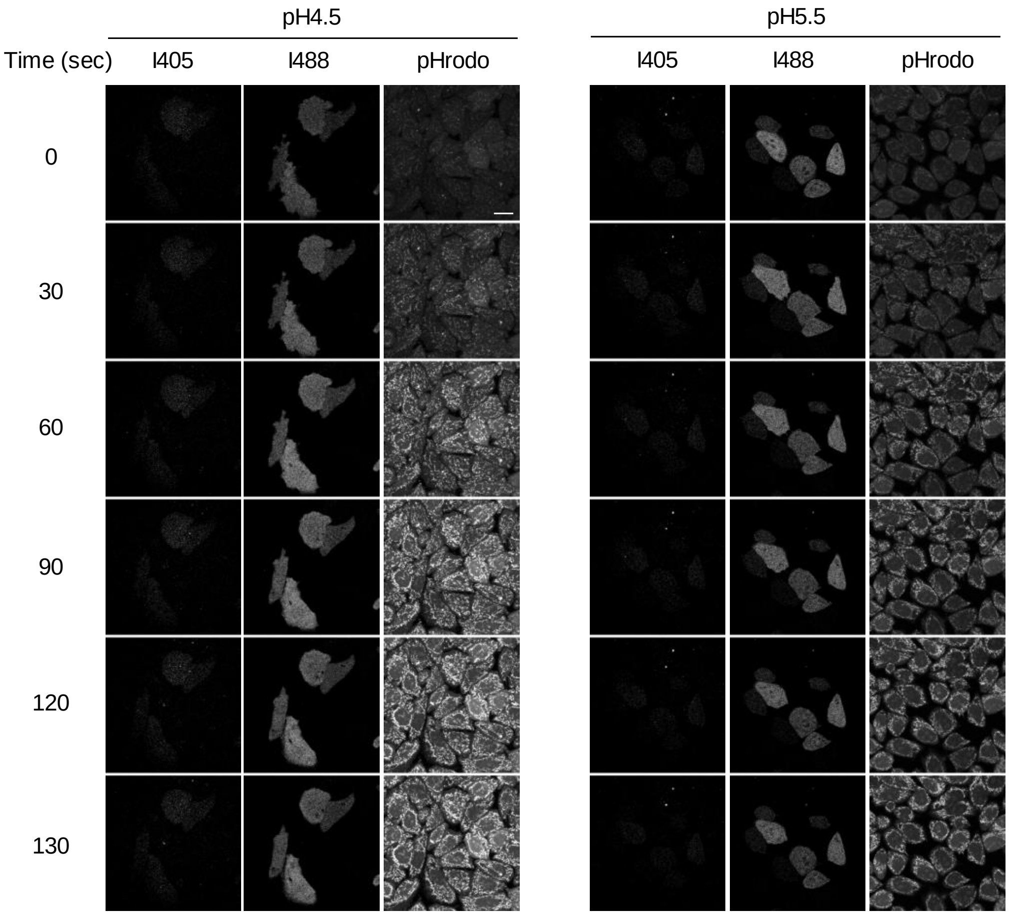

Scale bar, 20 μm

B-2

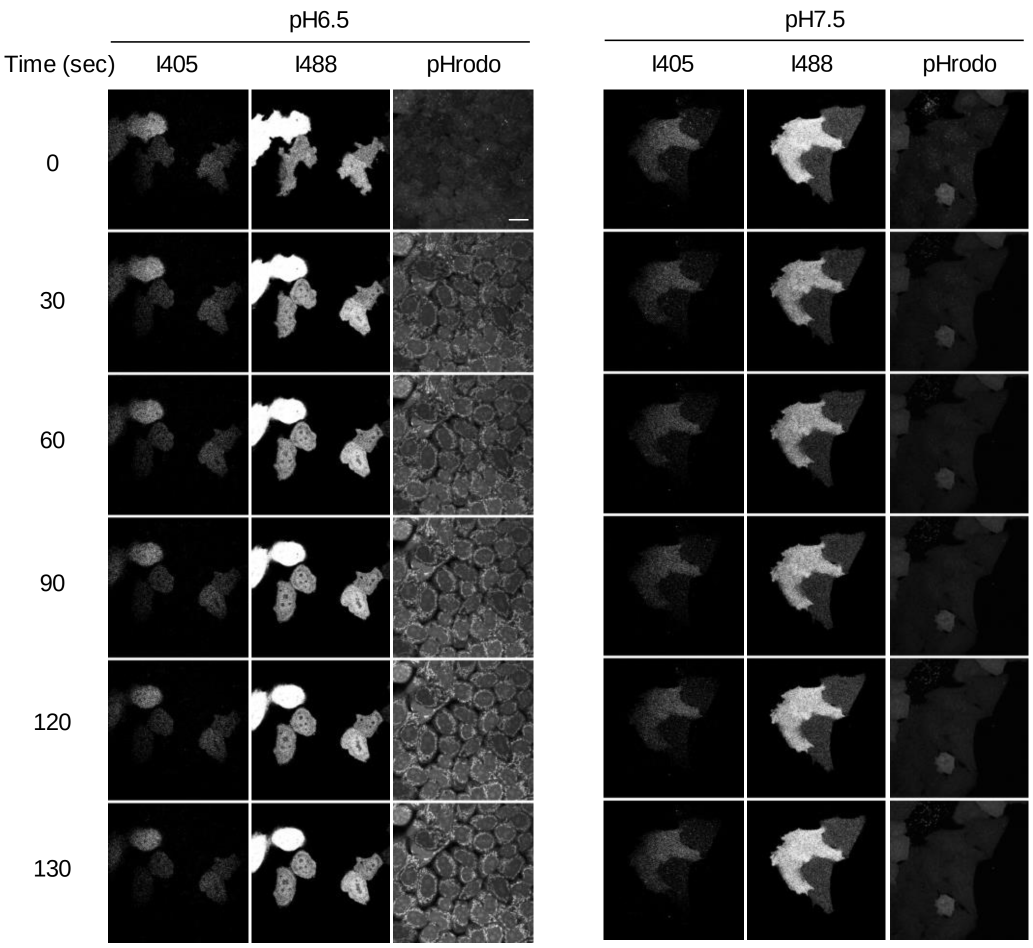

Scale bar, 20 μm

pHrodo Red AM Intracellular pH Indicator (P35372, Thermo Fisher scientific) was used for monitoring intracellular pH. Buffers with different pH (4.5, 5.5, 6.5, 7.5) and ionophore mixture (nigericin and valinomycin) were provided as Intracellular pH Calibration Buffer Kit (P35379, Thermo Fisher scientific). HeLa cells expressing Grx1-roGFP2 were loaded with pHrodo for 30 min and residual dyes were washed out by replenishing medium. Culture medium was then replaced with pH calibration buffer. As soon as adding ionophore mixture (10 μM nigericin and 10 μM valinomicin), time-lapse imaging was started. I405, I488 and pHrodo signals were simultaneously detected by confocal microscope. Quantitative analysis and original images are shown in A and B, respectively.

Supplementary Data 6: Superimposition of I405 and I488 channels.

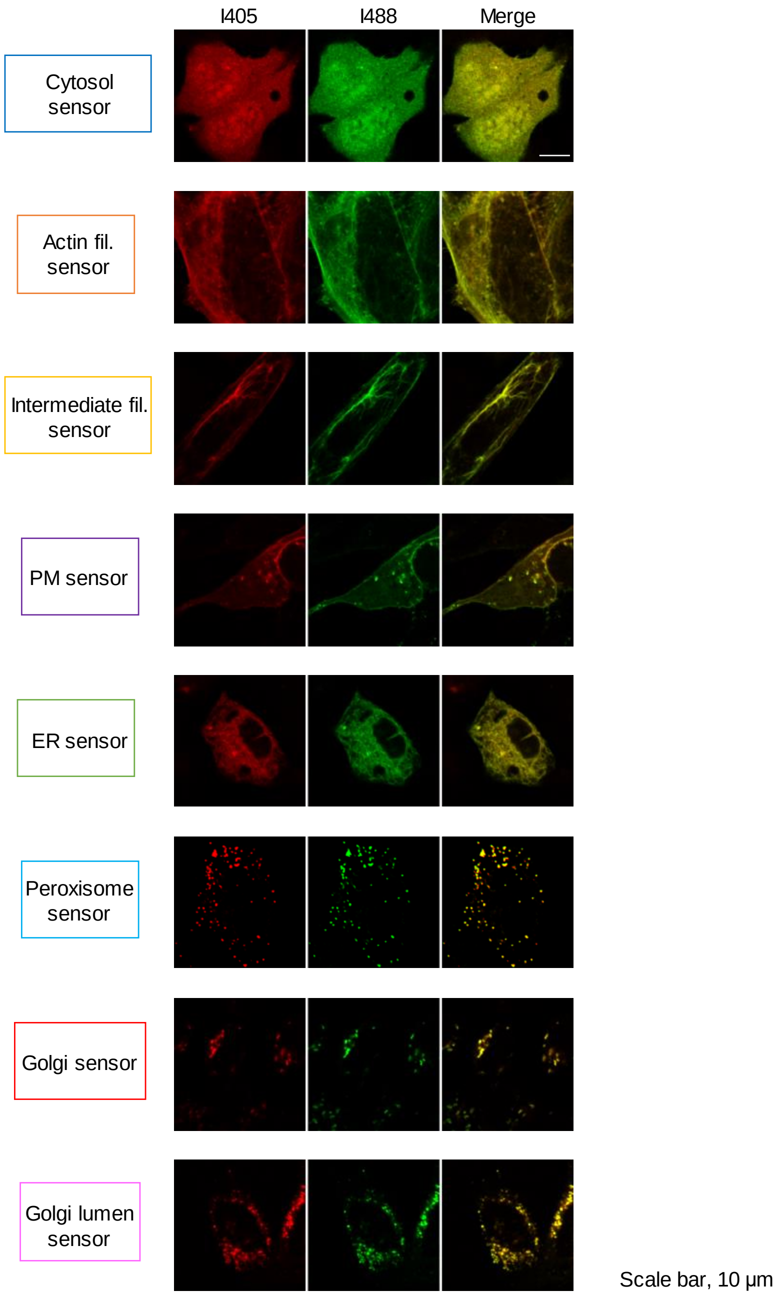

Supplementary Data 7: Original I405 and I488 images of fixed cells expressing Grx1-roGFP2 derivatives.

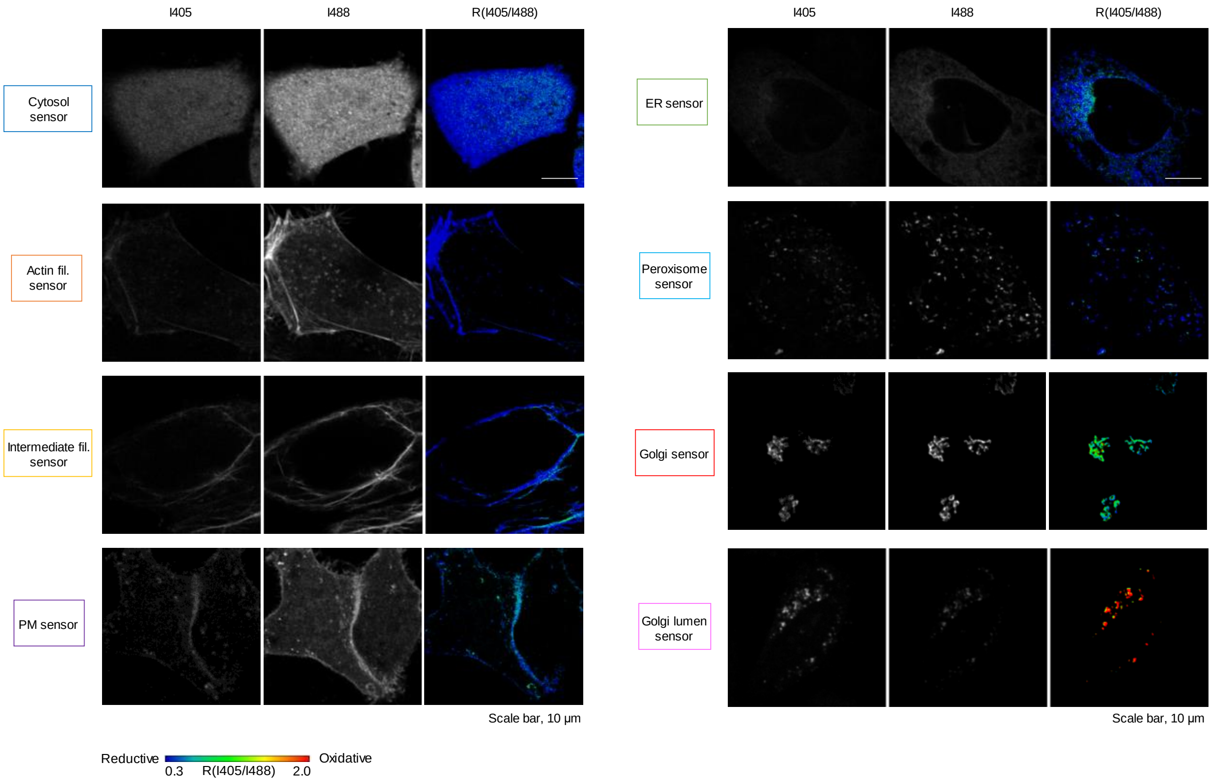

Supplementary Data 8: Topology assessment by Protease protection assay

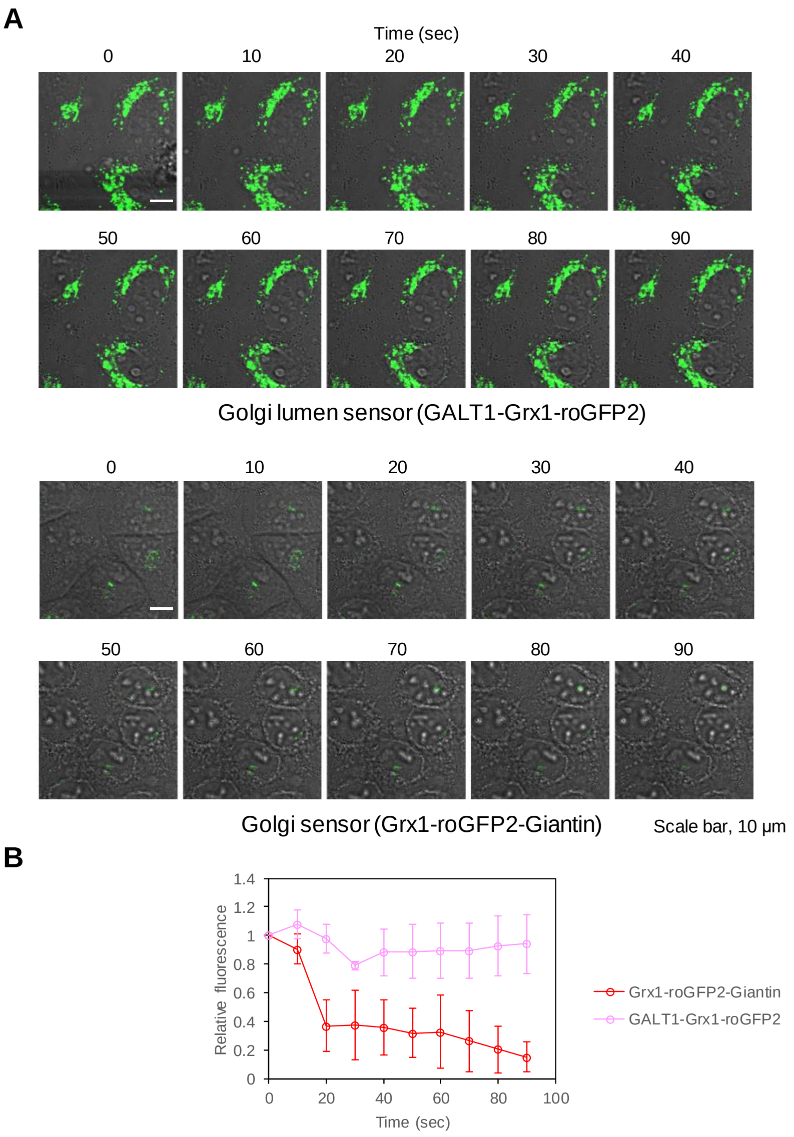

Membrane topologies of Grx1-roGFP2-Giantin and GALT1-Grx1-roGFP2 were experimentally compared based on fluorescence protease protection (FPP) assay. (a) Time-lapse images after addition of 50 μM digitonin and 50 μg/ml proteinase K. (b) Time-dependent decay of fluorescence from Grx1-roGFP2-Giantin. Fluorescence intensities were measured for multiple cells and normalized to the initial values.

Method: Membrane topologies of Grx1-roGFP2-Giantin and GALT1-Grx1-roGFP2 were experimentally compared based on fluorescence protease protection (FPP) assay [1]. According to previously established method[1], digitonin was used to preferentially permeabilize plasma membranes. Briefly, HeLa cells were cultured on collagen-coated 8-well glass bottom chambers (IWAKI 5232-008, AGC TECHNO GLASS, Japan) and transfected with 0.5 μg of plasmid DNA (either Grx1-roGFP2-Giantin and GALT1-Grx1-roGFP2) using Lipofectamine 3000 (Thermo Fisher scientific) according to manufacturer's instruction. Culture medium was replenished 24 h after transfection, and FPP assays were performed 48 h after transfection. Culture medium was replaced by serum-free DMEM and 50 μM digitonin and 50 μg/ml proteinase K were sequentially added to the cells, followed by time-lapse imaging using GFP channel. Experiments were repeated three times so that data for multiple cells were collected.

1. Lorenz, H.; Hailey, D.W.; Wunder, C.; Lippincott-Schwartz, J. The fluorescence protease protection (FPP) assay to determine protein localization and membrane topology. *Nature protocols* **2006**, *1*, 276-279, doi:10.1038/nprot.2006.42.
